# Autonomic Modulation in Duchenne Muscular Dystrophy during a Computer Task: A Prospective Control Trial

**DOI:** 10.1371/journal.pone.0169633

**Published:** 2017-01-24

**Authors:** Mayra Priscila Boscolo Alvarez, Talita Dias da Silva, Francis Meire Favero, Vitor Engrácia Valenti, Rodrigo Daminello Raimundo, Luiz Carlos Marques Vanderlei, David M. Garner, Carlos Bandeira de Mello Monteiro

**Affiliations:** 1 Physical Therapy, Speech and Occupational Therapy Department, School of Medicine, University of São Paulo, São Paulo, SP, Brazil; 2 Federal University of São Paulo, Paulista School of Medicine, São Paulo, SP, Brazil; 3 Federal University of São Paulo, Department of Neurology/Neurosurgery, Paulista School of Medicine, São Paulo, SP, Brazil; 4 Autonomic Nervous System Center Study, Speech Therapy Department Faculty of Sciences, Paulista State University (UNESP), Marília, SP, Brazil; 5 Laboratory Design and Scientific Writing, Department of Community Health, ABC Medical School, Santo André, SP, Brazil; 6 Department of Physiotherapy, Paulista State University (UNESP), Presidente Prudente, São Paulo, SP, Brazil; 7 Cardiorespiratory Research Group, Department of Biological and Medical Sciences, Faculty of Health and Life Sciences, Oxford Brookes University, Gipsy Lane, Oxford OX3 0BP, United Kingdom; 8 School of Arts, Sciences and Humanities, University of São Paulo, São Paulo, SP, Brazil; Faculty of Animal Sciences and Food Engineering, University of São Paulo, BRAZIL

## Abstract

**Introduction:**

Duchenne Muscular Dystrophy (DMD) is characterized by progressive muscle weakness that can lead to disability. Owing to functional difficulties faced by individuals with DMD, the use of assistive technology is essential to provide or facilitate functional abilities. In DMD, cardiac autonomic dysfunction has been reported in addition to musculoskeletal impairment. Consequently, the objective was to investigate acute cardiac autonomic responses, by Heart Rate Variability (HRV), during computer tasks in subjects with DMD.

**Method:**

HRV was assessed by linear and nonlinear methods, using the heart rate monitor Polar RS800CX chest strap Electrocardiographic measuring device. Then, 45 subjects were included in the group with DMD and 45 in the healthy Typical Development (TD) control group. They were assessed for twenty minutes at rest sitting, and five minutes after undergoing a task on the computer.

**Results:**

Individuals with DMD had a statistically significant lower parasympathetic cardiac modulation at rest when compared to the control group, which further declined when undergoing the tasks on the computer.

**Conclusion:**

DMD patients presented decreased HRV and exhibited greater intensity of cardiac autonomic responses during computer tasks characterized by vagal withdrawal when compared to the healthy TD control subjects.

## Introduction

Muscular dystrophies consist of a group of genetic disorders characterized by muscle weakness and atrophy [1, 2], particularly of early onset and of a progressive nature [3, 4].

Amongst all types of the muscular dystrophies, Duchenne Muscular Dystrophy (DMD) is considered the most widespread [5], with recessive genetic inheritance [3], and affecting approximately 1:3500 male births [6]. DMD occurs by a mutation of the gene encoding the dystrophin enzyme which is located on the short arm of the X chromosome [7] in the Xp21 region [1, 8]. DMD is categorized by the progressive loss of movement, which initially affects the lower limbs and then the upper limbs, with pseudo-hypertrophy of the affected muscles, interstitial increase of connective tissue and in the advanced stages significant increase of fat tissue in the muscles [9, 10].

On account of the functional difficulties presented by individuals with DMD; to enable the capability in social activities and performance; the practice of assistive technology or resources are needed. They achieve functional abilities of individuals with disabilities and thus promote greater independence and social inclusion [11]. According to Neistadt and Crepeau [12], assistive technology can be defined as any item or product, equipped for use, adapted or customized, that maintains or improves functional capabilities of individuals with a disability.

Recently the advances in computational assistive technology and the provision of rehabilitation programs using computer equipment during treatment allow the patient with DMD to undertake tasks in challenging situations by means of simple technology and achieving rapid responses. Additionally, it is possible to provide interactions with targets, through logical cognition and different reaction times associated with movement, allowing the repetition of muscle contractions and enhancing performance [13–20].

Moreover, the researched deficiencies in the musculoskeletal system [5] and cardiac autonomic dysfunction have been previously well researched in DMD [21, 22].

Thus, amongst the techniques applied to analyze the ANS, Heart Rate Variability (HRV) has emerged as a simple, reliable, inexpensive and non-invasive measure of the autonomic impulses. It represents one of the most promising quantitative markers of autonomic balance [23].

The wide use and cost-effectiveness of the technique and ease of data acquisition make HRV a capable choice for the interpretation of ANS functioning and a promising clinical tool to assess and identify physiological deficiencies [23]. Fluctuations in HRV patterns provide an early and sensitive diagnosis of the physiological behavior of the human body and health status of the individual [24].

Likewise, Thomas et al. [21] studied heart rate autonomic dysfunction in DMD. These authors assessed HRV in DMD and in a healthy control group by 24-hour Holter monitoring. They found that the control group demonstrated a higher maximum heart rate on Holter monitoring than in DMD patients. Dittrich et al. [22] assessed cardiac autonomic regulation in DMD, and the analytical value of the diagnostic procedures in clinical settings. Both of the abovementioned studies investigated the cardiac autonomic dysfunction in DMD. Consequently, the autonomic impairment is recognized for patients with DMD at rest. However, the effect of the computational task is not fully understood. At present, computer tasks are required for the groups independence and has been widely adopted in persons with DMD. It is especially important when assessing the influences on the autonomic system.

Whilst recent literature has revealed lesser HRV in DMD [21, 22], the specific cardiac autonomic response of this population is unclear when undergoing stimulation through computer tasks. There is little research on virtual technology procedures in DMD. Despite the use of computers in rehabilitation, we did not find any published research on the physiological changes that these tasks cause in individuals with DMD. To enable an understanding of these problems, this study evaluated these physiological adaptations by assessing the Autonomic Nervous System (ANS). Therefore, we aimed to investigate acute cardiac autonomic responses during computer tasks in individuals with DMD versus healthy people. If the HRV responses of DMD patients during computer tasks are improved comparing with the responses at rest, it provides new pathways for research using computational tasks that may be therapeutic by improving autonomic dysfunction in this group of subjects.

## Materials and Methods

### Participants

This is a prospective controlled trial. In this study were 90 age matched male subjects divided into equal groups with diagnosis of DMD and those healthy Typically Developed (TD) individuals without DMD. All individuals diagnosed with DMD were confirmed by molecular methods and/or protein expression in skeletal muscle.

Subjects were excluded with severely dilated myocardium, other associated diseases and individuals with changes in cognitive functions that would impede the simple cognition of commands in the proposed activities.

The research project (number 236/13) was approved by the research ethics committee of the University of São Paulo and undertaken after the signing of the Terms of Free and Informed Consent by the participants or legal guardian. Research participants aged 17 years or younger also submitted the research consent form.

To achieve the characterization of individuals with DMD, the Vignos scale was enforced [25]. This characterizes the disease severity according to pathological progression. They were classified from patient at stage 1 (walk and climb stairs without assistance) to 10 (permanently confined to bed).

### Data collection instruments

Data collection forms from the medical records were completed in individuals with DMD. It was used to obtain relevant information regarding patients' care, such as associated diseases and usage of medications.

A Premium Aneroid Sphygmomanometer (Model S82, Prestige Medical, Northridge, California, USA) and a BIC stethoscope (CBEMED, Itupeva, Brazil) were required to undertake systolic blood pressure (SBP) and diastolic blood pressure (DBP) measurements. Starting and final heart rate (HR) was verified by the investigator through their radial pulse and beats calculated for one minute.

HRV was recorded using the Polar RS800CX chest strap ECG measuring device (Polar Electro Oy, Kempele, Finland) previously validated to capture beat-to-beat HR (RR intervals), that represents the interval between each beat) [24].

### Collection procedures

SBP, DBP and HR measurements were assessed and recorded following the first minute of sitting and at the conclusion of twenty minutes of rest; then, before the start and at the end of the five minutes of the computer task.

Following the initial assessment, the capture strap was placed on the chest of volunteers and the HR receptor was placed on the wrist.

After strap placement and the computer screen startup, the individuals from both groups remained at rest and sitting in a chair (walkers, TD- and DMD-group) or in their own wheelchair (non-walkers, DMD-group), with spontaneous breathing for twenty minutes. Following this period, the computer screen was restarted, and the individuals remained seated with a notebook computer to enable them to perform a maze task on the computer for five minutes.

HRV was analyzed during two time periods: the period before (20 minutes) and then during the cognitive computer task (5 minutes).

**Fig 1:**
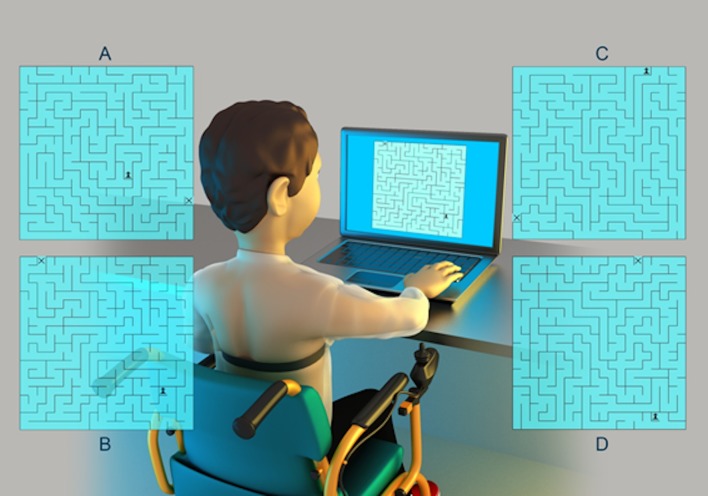
Example of Individual positioning during the maze task with the capture strap on the chest and mazes models used.

The computer task undertaken was a maze paradigm used for its cognitive requirements with ease and adaptability for use in individuals with DMD. To complete the tasks, the researchers selected a computer program developed by the Department of Mathematics of the Federal University of Rio Grande do Sul, presented by Souza et al. [26]. The task comprised of different maze designs, which had one correct pathway that could be negotiated. Fig 1 illustrates the experimental tasks.

The participants were evaluated individually in an appropriate room with a notebook computer, desk, chair (Fig 1) and the participation of an evaluator responsible for instruction and annotation of data collected.

Each individual was well positioned and the task was elucidated concurrently with the presentation of the maze, along which the individual should devise the path with the digital chess piece (pawn) character (pointed to, on the screen, by the evaluator) until the exit of the maze identified by an "x" (pointed to, on the screen, by the evaluator). The individual was instructed to perform the task as quickly as possible using the arrow buttons on the keyboard identified by arrows up, down, right and left.

A 20-by-20 cm maze was presented while the individuals undertook the task as many times as necessary to remain active at the computer for five minutes.

### Data analysis

The dependent variables (HRV indexes) were submitted to a 2 (group: DMD, TD) by 2 (Task: Rest, Computer) ANOVA with repeated measures on the last factor for each HRV index. Post-hoc comparisons were undertaken by Tukey-HSD (Honest Significant Difference) test (p < 0.05). For the physiologically independent variables such as Systolic Blood Pressure, Diastolic Blood Pressure and Heart Rate, the comparisons were made by using Student t test for unpaired data. The software package operated was SPSS, 20.0 (Chicago, Illinois, USA).

### HRV analysis

HRV analysis was completed under guidelines from the Task Force of the European Society of Cardiology and North American Society of Pacing and Electrophysiology [27]. The RR intervals were recorded using the portable Polar RS800CX heart rate (HR) monitor (Polar Electro, Finland) with a sampling rate of 1 kHz. They were downloaded to the Polar Precision Performance program (v.3.0). The software enabled the visualization of HR and the extraction of a cardiac period (RR interval; the variation of beat-to-beat separations) file in “txt” format. For analysis of HRV data in the sitting position, we analyzed 1000 consecutive RR intervals, and for HRV analysis for the computational task, the greatest number of consecutive RR intervals obtained was used, but with a minimum number of 256 RR intervals. Digital filtering complemented by manual filtering was performed to eliminate artifacts and only series with more than 95% of sinus beats were included in the study [28].

HRV analysis was assessed by linear methods, in the time (Dt) and frequency (Df) domains, and then by nonlinear methods (Poincaré plot). We chose to use linear and nonlinear methods, considering that both are shown to be complementary to each other, providing additional information [29].

### Linear methods

#### Time domain

In the Time domain Dt, the time interval between successive heart beat intervals, was determined by statistical and geometric methods [27].

The necessary statistical methods to assess ANS were SDNN (index of standard deviation of all normal-to-normal RR intervals), rMSSD (root mean square of successive differences between adjacent normal RR intervals), pNN50 (percentage of adjacent RR intervals with a difference longer than 50 milliseconds) [27].

For geometrical methods we enforced RR Tri (Total number of all NN intervals divided by the height of the histogram of all NN intervals) and TINN (baseline width of the minimum square difference triangular interpolation of the highest peak of the histogram of all NN intervals) [27].

#### Frequency Domain

For HRV analysis in Frequency domain, Df, low frequency (LF) and high frequency (HF) spectral components were appointed in absolute values of power (ms^2^) or in normalized units (n.u.). The ratio between these components in absolute values (LF/HF) represents the relative value of each spectral component in relation to the total potential minus the very low frequency (VLF) components [27].

The index Total (or Total Power) is the variance of NN intervals over the approximate temporal segment [27].

### Nonlinear methods

For HRV analysis by nonlinear methods, we applied the Poincaré plot (SD1 components—standard deviation of instantaneous beat-to-beat variability, SD2—standard deviation of long-term continuous RR intervals and relation SD1/SD2) [27].

The Poincaré plot enables each RR interval to be plotted against the next interval. For quantitative analysis of the plot, the indices for SD1, SD2 and the relation SD1/SD2 were calculated [30]. According to Hoshi et al., [31] “the Poincaré plot for heart rate variability analysis is a technique considered geometrical and non-linear, that can be used to assess the dynamics of heart rate variability by a representation of the values of each pair of R–R intervals into a simplified phase space that describes the system's evolution.”

For more information regarding the HRV indexes, see S1 File in the Supporting Information.

## Results

The age, anthropometric variables and medications taken by DMD group are stated in Table 1.

**Table 1:** Age, anthropometric variables within the groups (by mean ± standard deviation) and the cardiac medication for DMD-group.

**Variable**	**TD-group**	**DMD-group**	**p**
Age (years)	15.4 ± 2.8	15.4 ± 2.9	0.455
Height (m)	1.68 ± 0.12	1.56 ± 0.17	<0.001
Mass (kg)	63.2 ± 15.5	55.84 ± 17.9	0.013
BMI (kg/m^2^)	20.04 ± 3.72	22.42 ± 4.71	0.331
**Medication on DMD-group**		**Number of patients (%)**	
Beta-blockers		13 (28.89)	
ACE-inhibitor		5 (11.11)	
Beta-blockers + ACE-inhibitors		20 (44.44)	
No medication		7 (15.56)	

TD: Typical Development; DMD: Duchenne Muscular Dystrophy; BMI: body mass index; m: meters; kg: kilograms; kg/m^2^: kilograms per square meter; ACE-inhibitors: angiotensin-converting enzyme inhibitors.

Individuals with DMD were classified by Vignos Scale, as described in Table 2.

**Table 2:** Description individuals with DMD by Vignos Scale.

Vignos scale		Number of patients
1	Walks and climbs stairs without assistance.	4
2	Walks and climbs stairs with aid of railing.	4
3	Walks and climbs stairs slowly with aid of railing (more than 25 seconds for eight standard steps).	1
4	Walks unassisted and rises from chair; cannot climb stairs.	1
5	Walks unassisted; cannot rise from chair; cannot climb stairs.	0
6	Walks only with assistance or walks independently with leg braces.	0
7	Walks in leg braces, but requires assistance for balance.	19
8	Maintains standing with leg braces, but is unable to walk even with assistance.	14
9	In wheelchair.	2
10	Confined to bed.	0

### Heart rate variability

#### Time domain

For statistical methods, concerning the mean RR interval there was no effect of the computer Task. However, there was an interaction between Task and Group (F _1,88_ = 4.44, p = 0.038, η^2^ = 0.05). The post-hoc test illustrated that there was difference between tasks just for TD group (p = 0.009) (Table 3). There was a main effect for Group (F _1,88_ = 24.5, p< 0.001, η^2^ = 0.22). This implied that the TD group mean RR interval was higher than in the DMD group.

**Table 3:** Time domain indices of HRV at rest seated and during cognitive task in the TD and DMD groups.

Index	TD		DMD	
	Rest seated	Cognitive task	Rest seated	Cognitive task
Statistical methods				
Mean RR (ms)	757.3 ± 88.6*	779.13 ± 105.7*	675.06 ± 90.99	672.40 ± 91.72
SDNN (ms)	79.41 ± 33.41*	63.47 ± 34.70*	54.40 ± 25.06*	36.72 ± 16.94*
RMSSD (ms)	53.55 ± 29.49*	52.23 ± 32.48*	37.21 ± 20.98*	31.23 ± 20.36*
pNN50 (%)	25.48 ± 16.27	27.79 ± 21.16	15.82 ± 15.89*	12.35 ± 14.65*		
Geometrical methods				
RR Tri	19.38 ± 8.05	14.22 ± 4.61	14.20 ± 6.32	9.51 ± 3.29
TINN (ms)	355.67 ± 143.14	255.56 ± 89.69	262.89 ± 113.61	165.44 ± 67.86

TD: Typical Development; DMD: Duchenne Muscular Dystrophy. SDNN: standard deviation of normal-to-normal RR intervals; pNN50: the percentage of adjacent RR intervals with a difference of duration greater than 50 ms; RMSSD: root-mean square of differences between adjacent normal RR intervals in a time interval; RR Tri: triangular index; TINN: triangular interpolation of RR intervals; ms: milliseconds.

*p<0.05 cognitive task vs rest seated in each group. There was significant difference between groups for all time domain indexes.

Regarding SDNN there was an effect for Task (F _1,88_ = 51.3, p< 0.001, η^2^ = 0.37), but no interaction between Task and Group. A Main effect for Group (F _1,88_ = 21.9, p< 0.001, η^2^ = 0.20) was found. In the TD group the SDNN was higher than in the DMD group.

With respect to rMSSD, similarly to SDNN, there was an effect for Task (F _1,88_ = 5.11, p = 0.026, η^2^ = 0.06), but no interaction between Task and Group. A Main effect for Group (F _1,88_ = 12.2, p = 0.001, η^2^ = 0.12) remained. In the TD group, the rMSSD was higher than in the DMD group.

For pNN50 there was no effect for Task. Yet, there was an interaction between Task and Group (F _1,88_ = 5.44, p = 0.022, η^2^ = 0.06). The post-hoc test exhibited that there was difference between tasks, but only for the DMD group (p = 0.047). Additionally, there was a main effect for Group (F _1,88_ = 13.5, p< 0.001, η^2^ = 0.13). We observed that the TD group pNN50 was greater than in the DMD group.

In the geometrical methods, for RR Triangular Index there was an effect for Task (F _1,88_ = 77.2, p< 0.001, η^2^ = 0.47), but no interaction between Task and Group (see values in Table 3). A Main effect for Group (F _1,88_ = 20.2, p< 0.001, η^2^ = 0.19) was found. In the TD group the RR Triangular Index was higher than in the DMD group (Table 3).

Similarly, for TINN there was an effect for Task (F _1,88_ = 56.4, p< 0.001, η^2^ = 0.39), but no interaction between Task and Group. A Main effect for Group (F _1,88_ = 24.7, p< 0.001, η^2^ = 0.22) was found. In the TD group the TINN was higher than in the DMD group.

#### Frequency domain

Regarding the LF (ms^2^) there was an effect for Task (F _1,88_ = 55.0, p< 0.001, η^2^ = 0.39), but no interaction between Task and Group (see values in Table 4). A Main effect for Group (F _1,88_ = 12.6, p = 0.001, η^2^ = 0.13) was found. In the TD group the LF (ms^2^) was higher than in the DMD group (Table 4).

**Table 4:** Frequency domain indices of HRV at rest seated and during cognitive task in the TD and DMD groups.

Index	TD		DMD	
	Rest seated	Cognitive task	Rest seated	Cognitive task
LF (ms^2^)	2039.69 ± 1288.77	1184.84 ± 1314.29	1191.11 ± 1148.62	510.47 ± 625.12
LF (n.u.)	65.97 ± 14.82	58.04 ± 17.53	65.44 ± 11.63	58.35 ± 16.89
HF (ms^2^)	1316.04 ± 1552.74	1150.36 ± 1607.58	702.18 ± 704.87	472.31 ± 703.89
HF (n.u.)	34.03 ± 14.82	41.96 ± 17.53	34.56 ± 11.64	41.65 ± 16.89
Total (ms^2^)	6652.91 ± 6214.01	4236.51 ± 5617.50	3111.96 ± 2785.06	1412.47 ± 1554.07
LF/HF (ms^2^)	2.73 ± 2.43	2.31 ± 3.32	2.26 ± 1.18	1.87 ± 1.27

TD: Typical Development; DMD: Duchenne Muscular Dystrophy. LF: low frequency; HF: high frequency; LF/HF: low frequency/ high frequency ratio; ms: milliseconds; ms^2^: milliseconds squared; n.u.: normalized units.

There was significant difference between cognitive task vs rest seated in both groups for all frequency domain indexes. For LF, HF and LF/HF there was no significant difference between groups.

Regarding the LF (normalized units), there was an effect for Task (F _1,88_ = 26.2, p< 0.001, η^2^ = 0.23), but no interaction between Task and Group. There was no Main effect for Group.

With respect to the HF (ms^2^), similarly to LF (ms^2^) there was an effect for Task (F _1,88_ = 7.29, p = 0.008, η^2^ = 0.08), but no interaction between Task and Group. A Main effect for Group (F _1,88_ = 12.6, p = 0.001, η^2^ = 0.13) was found. In the TD group the HF (ms^2^) was higher than in the DMD group.

In relation to the HF (normalized units), there was an effect for Task (F _1,88_ = 26.2, p< 0.001, η^2^ = 0.23), but no interaction between Task and Group. There was no Main effect for Group.

For Total power there was an effect for Task (F _1,88_ = 24.9, p< 0.001, η^2^ = 0.22), but no interaction between Task and Group. A Main effect for Group (F _1,88_ = 14.0, p< 0.001, η^2^ = 0.14) was found. In the TD group the Total was higher than in the DMD group.

When we assess the LF/HF, there was an effect for Task (F _1,88_ = 4.10, p = 0.046, η^2^ = 0.04), but no interaction between Task and Group. There was no Main effect for Group.

#### Nonlinear methods

Concerning the SD1 there was an effect for Task (F _1,88_ = 5.24, p = 0.025, η^2^ = 0.06), but no interaction between Task and Group. A Main effect for Group (F _1,88_ = 12.2, p = 0.001, η^2^ = 0.12) was found. In the TD group the SD1 was higher than in the DMD group (Table 5).

**Table 5:** Mean and standard deviation from nonlinear indices in both groups.

Index	TD		DMD	
	Rest seated	Cognitive task	Rest seated	Cognitive task
SD1 (ms)	37.88 ± 20.85	36.98 ± 23.01	26.33 ± 14.84	22.11 ± 14.41
SD2 (ms)	105.21 ± 43.69	80.73 ± 45.27	71.99 ± 32.86	46.46 ± 20.24
SD1/SD2 (ms)	0.35 ± 0.11	0.46 ± 0.18	0.36 ± 0.10	0.45 ± 0.16

TD: Typical Development; DMD: Duchenne Muscular Dystrophy; SD1: standard deviation of instantaneous beat-to-beat variability; SD2: standard deviation of long-term continuous RR intervals; ms: milliseconds.

There was significant difference between cognitive task vs rest seated in both groups for all frequency domain indexes. For SD1/SD2 there was no significant difference between groups.

Regarding the SD2 there was an effect for Task (F _1,88_ = 54.1, p< 0.001, η^2^ = 0.38), but no interaction between Task and Group. A Main effect for Group (F _1,88_ = 23.4, p< 0.001, η^2^ = 0.21) remained. In the TD group the SD2 was higher than in the DMD group.

**Fig 2:**
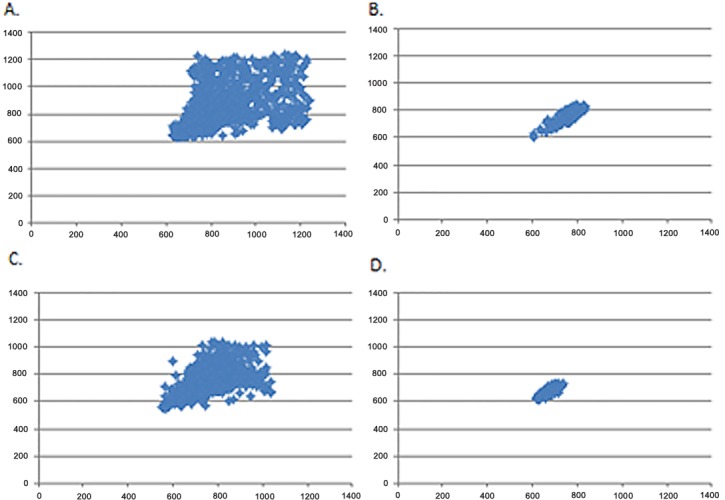
Visual pattern of the Poincaré plot observed in one subject before (A) and during cognitive computer task (B) in the TD group and in one subject before (C) and during cognitive computer task (D) in the DMD group.

With respect to the SD1/SD2 ratio, there was an effect for Task (F _1,88_ = 19.8, p< 0.001, η^2^ = 0.18), but no interaction between Task and Group. There was no Main effect for Group. Fig 2 represents the visual pattern of the Poincaré plot observed in one subject from each group, before and after cognitive task.

Table 6 illustrates HR, SBP and DBP values for the start and completion of the cognitive task. We observed no significant responses for those variables during the cognitive computer task.

**Table 6:** Mean and standard error for HR (bpm), SBP (mmHg) and DBP (mmHg) initial and final in the cognitive task.

Variable	TD			DMD		
	Initial	Final	p	Initial	Final	p
HR	77.3±2.4	79.1±1.6	0.094	89.7±1.7	89.2±1.9	0.599
SBP	116.2±1.9	122.4±1.8	0.002	104.3±1.9	102.1±1.9	0.191
DBP	72.5±2.0	76.1±1.6	0.029	72.3±1.6	71.5±1.6	0.657

TD: Typical Development; DMD: Duchenne Muscular Dystrophy, HR: Heart rate, SBP: Systolic Blood Pressure, DBP: Diastolic Blood Pressure.

## Discussion

We evaluated HRV responses induced by a computer task in DMD patients. As expected we discovered significant basal HRV differences between the groups, indicating that DMD causes a decreased HRV. We also obtained a statistically significant greater intensity of responses in the DMD group compared to the control group, since parasympathetic withdrawal during the task achieved higher responses in the DMD patients.

The ANS consists of two components, sympathetic and parasympathetic. To maintain physiological homeostasis, the sympathetic nervous system (SNS) and parasympathetic nervous system (PNS) perform antagonistically. In response to stress, exercise and heart disease there is a sympathetic stimulation which causes an elevation in HR. To provide a regulatory balance in physiological autonomic function, the parasympathetic modulation operates by decreasing the HR [32]. Consequently, ANS act together in coordinating visceral activities, adapting the functioning of each organ to circumstances to which the body is subjected.

Considering at rest HRV, this study found that the statistical differences between groups indicate lower parasympathetic modulation (rMSSD, pNN50, HF, SD1) and overall HRV (SDNN, LF, RRtri, TINN, SD2) in the DMD group, which reflects a lower ANS adaptive capacity due to pathological impairment.

Our results are supported by Dhargave et al. [33], who evaluated 124 patients with DMD and compared them with 50 age matched individuals in the supine position, and attained a reduction in autonomic regulation in the DMD group, with decreased PNS modulation and increased sympathetic predominance.

The cited studies [34, 35] evaluated patients with DMD and documented an increase in sympathetic modulation with reduced parasympathetic modulation at various pathological stages. This suggested that ANS involvement occurs in the early stages of DMD and likely due to progressive inactivity and neglect of physical fitness. Accordingly, the authors concluded that with advancing pathology, a secondary autonomic imbalance of cardiopulmonary dysfunction, along with progressive inactivity and lack of conditioning might increase the inherent autonomic abnormalities.

In 2001, Lanza [36] established lower HRV in DMD patients compared with the control group of healthy individuals. The data of Inoue et al. [37] illustrated that the autonomic abnormalities in the patients with DMD are characterized by a significant decrease in parasympathetic modulation and a significant increase in sympathetic modulation.

Whilst performing the computer tasks, this study reports that parasympathetic modulation is diminished in both groups. However, HRV responses were more intense in the DMD patients, since the pNN50 index was significant for the DMD group but no significant responses were found in the control group. Furthermore, the Poincaré plot demonstrated decreased HRV while performing the computer tasks when compared to at rest in both groups. However, the decrease was greater in the DMD group.

The quantitative analysis of the Poincaré plot provides analysis of chaotic behavior of heart rate dynamics [31]. In this context, we achieve an understanding of nonlinear analysis of HRV. If only linear methods are applied to RR intervals some information may be lost. Thus, indicating that the traditional time and frequency domains analysis are mostly insufficient to characterize the complexity of the heart rate dynamics [38].

Also, nonlinear analysis of HRV does not assess responses associated with the quantification of variability. It only provides the quality and correlation properties of the signal [39]. Previous studies have demonstrated nonlinear methods as clinically important to interpretation of pathological mechanisms related to HRV. So the nonlinear method provides extra information to linear methods alone [40, 41].

The tasks performed in this current study are identical in both groups. Nevertheless, due to the presence of progressive muscle weakness which is characteristic of the disease, we assert that muscular effort was greater in the DMD group of patients. This included patients with various degrees of severity, ranked from 1 to 9 on a scale of Vignos. The greatest effort exerted during the task led to the reduction of parasympathetic modulation, necessary to maintain homeostasis of the body to the stimulus.

We reflect that the impaired cardiac autonomic modulation in DMD patients hindered their ANS response to cognitive stimulation, such as the computer task in this study.

Thus, the relationship between HRV and respiratory function in DMD might be explained by our data. Significant correlation was achieved between forced vital capacity and HRV indices [36]. Therefore, we hypothesized that the reduced respiratory strength in DMD patients caused by impaired muscle function is implicit in higher HRV responses to the computer task. Alternatively, it is important to mention that the authors noted moderate correlation (r = 0.3), suggestive that other factors are involved in the autonomic change.

The role of dystrophin in cardiac autonomic modulation could be involved in the elevation of HRV responses to cognitive computer tasks [42]. Dysfunction in the cognitive abilities of DMD is also suggested to be involved in changes in HRV responses to computer tasks. DMD is associated with behavioral and cognitive disabilities leading to impaired intellectual disabilities and lowered academic achievement [43].

This study suggests cardiac autonomic modulation data, useful for clinical practice by indicating that the use of computer tasks can support the functional capacities through training and competence of the ANS. Yet, the computational tasks must be performed under supervision and care taken to avoid psychological overload and exacerbation to the ANS.

The recent literature has found reduced HRV in DMD [21,22], and it is well known that HRV in DMD is reduced, possibly leading to cardiac [34–36] or respiratory [36–44] failure. As the computational task induces sympathetic primacy it may be applied as a tool for improvement of heart rate autonomic modulation.

This study has limitations that should be recognized: (1) Patients were included in the study that continued to use beta-blockers and angiotensin-converting enzyme (ACE) inhibitors. Despite the interference that medication could cause to autonomic functions. These medications are frequently used and their cessation is not medically feasible. Inoue et al. [37] reported that by limiting the study without the evaluation of patients taking medication for congestive heart failure, only individuals with milder form of the disease participated in the study; (2) The inclusion of patients with varying degrees of pathology (Vignos scale 1–9). However, the principle of obtaining HRV data at different stages of the disease is vital to better characterize the population.

## Conclusion

DMD patients presented decreased HRV and exhibited greater intensity of cardiac autonomic responses during computer tasks characterized by vagal withdrawal when compared to the healthy typically developed control subjects.

## Supporting Information

S1 File Supplementary material about HRV. (PDF)
