# Supplementary material for: Autonomic Modulation in Duchenne Muscular Dystrophy during a Computer Task: A Prospective Control Trial
Source: PLoS One. 2017 Jan 24;12(1):e0169633. doi: 10.1371/journal.pone.0169633 (PMC5261738; doi:10.1371/journal.pone.0169633)
Supplement: S1 File — (PDF) [file pone.0169633.s001.pdf]

Supplementary material

HRV analysis

The software applied for analysis of linear and nonlinear methods was Kubios HRV (University of Eastern Finland, Joensuu, Finland).

Time Domain

RMSSD, which consists of parasympathetic modulation, is defined and shown below in the equation (Marães et al., 2003):

$$RMSSD = \sqrt{\frac{\sum_{i-1}^{N-1}(RR_i - RR_{i+1})^2}{N-1}}$$

Where: RR = RR intervals; N = number of RR intervals in the selected data series.

The pNN50 index is a sensitive marker and easily interpretable of autonomic modulation. The pNN50 index is defined as the percentage of successive differences of the RR interval whose absolute value exceeds 50ms (TFESC & NASPE, 1996).

SDNN, which reflects the involvement of both branches of the ANS, is the mean RR interval, standard deviation of all normal-to-normal RR intervals, expressed in milliseconds (Pumprla et al., 2002; Bittencourt et al., 2005).

The geometric methods applied to analyze D*t* in this study were: RRtri (triangular index) and TINN (triangular interpolation of RR intervals).

RRtri assesses the full distribution of RR intervals divided by the distribution of the maximum density (histogram height). TINN corresponds to the width of baseline distribution, measured as a base of one triangle (TFESC & NASPE, 1996).

Frequency Domain

The methods in D*f* permit the individual analysis of the ANS in various physiological and pathological conditions and its relationship to other systems that also interfere with HRV (TFESC & NASPE, 1996).

Nonlinear Methods

Poincaré Plot

Qualitative analysis of the plot was made through the analysis of the figures formed by its attractor, which were described by Tulppo et al. (1998) in which: 1) Figure in which an increase in dispersion of RR intervals is observed with increased intervals, characteristic of a normal plot; 2) Figure with a small overall beat-to-beat dispersion and without long run increased dispersion of RR intervals; 3) Complex or parabolic figure, in which two or more distinct ends are separated from the main body of the plot, with at least three points included in each end.
